# Applying Multiple Criteria Decision Analysis (MCDA) in the context of HTA: an experimental case study on metastatic colorectal cancer

**DOI:** 10.1186/2052-3211-8-S1-O8

**Published:** 2015-10-05

**Authors:** Aris Angelis, Panos Kanavos

**Affiliations:** 1 London School of Economics and Political Science, London, WC2A 2AE, UK

## Problem statement

There is increased recognition that economic evaluation has limitations because it does not capture a number of important dimensions of value. This may lead to a lack of comprehensiveness and a lack of transparency.

## Objectives

Past research has indicated that Multiple Criteria Decision Analysis (MCDA) could be used as an alternative methodology for assessing the value of new medical technologies in the context of Health Technology Assessment (HTA). The objective of this study is to apply in practice an MCDA framework for the value assessment of a set of therapeutic options in metastatic colorectal cancer through a simulation exercise based on MCDA principles.

## Policies targeted

A process to inform the value assessment of new medical technologies which, in turn, can help determine coverage decisions and possibly pricing mechanisms.

## Stakeholders

All stages of the framework were informed by extensive stakeholder engagement through their participation at a decision conference workshop. Stakeholders (13 in total) included health care professionals (e.g. clinical experts), methodology experts (e.g. health economists) and patient advocates (e.g. patients and carers), while adopting the perspective of a decision-making HTA body.

## Region covered

The context of the study relates to England and Wales at a national level (EURO region), but it can be applicable across all WHO regions and levels.

## Methods

Study design: experimental case study

Time period: January 2015-May 2015.

Setting: to inform coverage decisions for cancer medicines used in inpatient settings

Interventions: application of a pre-existing methodological framework and experimental case study through a decision conference.

## Results

Value parameters considered included therapeutic, safety, innovation and socioeconomic criteria. Three alternative treatments were ranked based on their overall value, using value scores reflecting their performance across all the criteria while considering their relative importance, as informed through stakeholders' preferences. Simulation of payer's resource allocation decisions on the coverage of the options were made on value for money grounds through the use of a “cost-per-unit of value” metric.

## Conclusions

MCDA possesses the prerequisites of a value-based assessment methodological framework. The multiplicity of criteria that can be incorporated to assess value, the weights that can be applied to the criteria and the stakeholders' involvement across every stage, all of which are fully transparent, provide a unique combination of broadness, resilience and inclusiveness making it an ideal decision-making tool.

